# Supplementary material for: Glucomannan engineering highlights roles of galactosyl modification in fine-tuning cellulose-glucomannan interaction in Arabidopsis cell walls
Source: Nat Commun. 2025 Jan 31;16:1235. doi: 10.1038/s41467-025-56626-y (PMC11785759; doi:10.1038/s41467-025-56626-y)
Supplement: Supplementary file 3 — Description of Additional Supplementary Files [file 41467_2025_56626_MOESM3_ESM.pdf]

Description of Additional Supplementary Files

File Name: Supplementary Data 1
Description: Chemical list.

File Name: Supplementary Data 2
Description: GT34 amino acid sequences used for phylogenetic tree in Supplementary Figure 1.

File Name: Supplementary Data 3
Description: Primers that used in this work.

File Name: Supplementary Data 4
Description: Synthetic genes and Golden Gate parts used in this work.

File Name: Supplementary Data 5
Description: Statistics analysis on monosaccharides composition of AIR measured by sulphuric acid hydrolysis and HPAEC-PAD.

File Name: Supplementary Data 6
Description: Statistics analysis on monosaccharides composition of cell wall fractions measured by TFA hydrolysis and HPAEC-PAD.

File Name: Supplementary Data 7
Description: ^{13}C solid-state NMR chemical shift assignments of cell wall components in wild-type Arabidopsis inflorescence stem.
