# Supplementary material for: Glucomannan engineering highlights roles of galactosyl modification in fine-tuning cellulose-glucomannan interaction in Arabidopsis cell walls
Source: Nat Commun. 2025 Jan 31;16:1235. doi: 10.1038/s41467-025-56626-y (PMC11785759; doi:10.1038/s41467-025-56626-y)
Supplement: Supplementary file 11 — Reporting Summary [file 41467_2025_56626_MOESM11_ESM.pdf]

nature portfolio

Corresponding author(s): Paul Duree

Last updated by author(s): Jan 24, 2025

Reporting Summary

Nature Portfolio wishes to improve the reproducibility of the work that we publish. This form provides structure for consistency and transparency in reporting. For further information on Nature Portfolio policies, see our Editorial Policies and the Editorial Policy Checklist.

Statistics

For all statistical analyses, confirm that the following items are present in the figure legend, table legend, main text, or Methods section.

n/a	Confirmed	
☐	☒	The exact sample size (*n*) for each experimental group/condition, given as a discrete number and unit of measurement
☐	☒	A statement on whether measurements were taken from distinct samples or whether the same sample was measured repeatedly
☐	☒	The statistical test(s) used AND whether they are one- or two-sided<br>*Only common tests should be described solely by name; describe more complex techniques in the Methods section.*
☒	☐	A description of all covariates tested
☒	☐	A description of any assumptions or corrections, such as tests of normality and adjustment for multiple comparisons
☐	☒	A full description of the statistical parameters including central tendency (e.g. means) or other basic estimates (e.g. regression coefficient) AND variation (e.g. standard deviation) or associated estimates of uncertainty (e.g. confidence intervals)
☐	☒	For null hypothesis testing, the test statistic (e.g. *F*, *t*, *r*) with confidence intervals, effect sizes, degrees of freedom and *P* value noted<br>*Give P values as exact values whenever suitable.*
☒	☐	For Bayesian analysis, information on the choice of priors and Markov chain Monte Carlo settings
☒	☐	For hierarchical and complex designs, identification of the appropriate level for tests and full reporting of outcomes
☒	☐	Estimates of effect sizes (e.g. Cohen's *d*, Pearson's *r*), indicating how they were calculated

Our web collection on statistics for biologists contains articles on many of the points above.

Software and code

Policy information about availability of computer code

Data collection	TOPSPIN 4.1.3 (Bruker) was used for NMR data collection. Chromeleon™ v6.80 was used to collect HPAEC-PAD data.
Data analysis	MEGA X (version 10.2.6) was used to generate amino acid sequence alignment and phylogenetic analysis. Amino acid sequence alignment was visualised by ESPript 3.0 web (https://espript.ibcp.fr). DeepTMHMM ver 1.06 (https://dtu.biolib.com/DeepTMHMM) was used to predict a transmemebrane domain of MAGT proteins. ColabFold v1.5.5 (https://colab.research.google.com/github/sokrypton/ColabFold/blob/main/AlphaFold2.ipynb) was used to predict MAGT protein structures and Pymol version 2.5 was used for visualisation and creating images. Image J version 1.53q was used to measure the size of Arabidopsis seed mucilage. Microsoft® Excel® (Microsoft 365 MSO Version 2408) and GraphPad Prism10 version 10.1.2 were used for data analysis and statistical analysis. TOPSPIN 4.1.3 (Bruker) was used for NMR data analysis.

For manuscripts utilizing custom algorithms or software that are central to the research but not yet described in published literature, software must be made available to editors and reviewers. We strongly encourage code deposition in a community repository (e.g. GitHub). See the Nature Portfolio guidelines for submitting code & software for further information.

Data

Policy information about availability of data

All manuscripts must include a data availability statement. This statement should provide the following information, where applicable:

- Accession codes, unique identifiers, or web links for publicly available datasets
- A description of any restrictions on data availability
- For clinical datasets or third party data, please ensure that the statement adheres to our policy

Amino acid sequences of GT34 used for phylogenetic analysis are available from the NCBI protein database (https://www.ncbi.nlm.nih.gov/) and PLAZA database (https://bioinformatics.psb.ugent.be/plaza/) and all protein sequences used in this work were provided in Supplementary Data 4. Source Data are provided with this paper. The unprocessed solid-state NMR data are available in BMRB database under the entry ID, BMRbig111 [https://bmrbig.org/released/bmrbig111].
6BSW [https://doi.org/10.2210/pdb6BSW/pdb]
SALK_061576 [https://www.arabidopsis.org/germplasm?key=4664164]
At2g22900 [https://bioinformatics.psb.ugent.be/plaza/versions/plaza_v5_dicots/genes/view/AT2G22900]
At4g37690 [https://bioinformatics.psb.ugent.be/plaza/versions/plaza_v5_dicots/genes/view/AT4G37690]
DAA64590.1 [https://www.ncbi.nlm.nih.gov/protein/DAA64590.1]
Q564G7.1 [https://www.ncbi.nlm.nih.gov/protein/Q564G7.1]

Research involving human participants, their data, or biological material

Policy information about studies with human participants or human data. See also policy information about sex, gender (identity/presentation), and sexual orientation and race, ethnicity and racism.

Reporting on sex and gender	*Use the terms sex (biological attribute) and gender (shaped by social and cultural circumstances) carefully in order to avoid confusing both terms. Indicate if findings apply to only one sex or gender; describe whether sex and gender were considered in study design; whether sex and/or gender was determined based on self-reporting or assigned and methods used. Provide in the source data disaggregated sex and gender data, where this information has been collected, and if consent has been obtained for sharing of individual-level data; provide overall numbers in this Reporting Summary. Please state if this information has not been collected. Report sex- and gender-based analyses where performed, justify reasons for lack of sex- and gender-based analysis.*
Reporting on race, ethnicity, or other socially relevant groupings	*Please specify the socially constructed or socially relevant categorization variable(s) used in your manuscript and explain why they were used. Please note that such variables should not be used as proxies for other socially constructed/relevant variables (for example, race or ethnicity should not be used as a proxy for socioeconomic status). Provide clear definitions of the relevant terms used, how they were provided (by the participants/respondents, the researchers, or third parties), and the method(s) used to classify people into the different categories (e.g. self-report, census or administrative data, social media data, etc.) Please provide details about how you controlled for confounding variables in your analyses.*
Population characteristics	*Describe the covariate-relevant population characteristics of the human research participants (e.g. age, genotypic information, past and current diagnosis and treatment categories). If you filled out the behavioural & social sciences study design questions and have nothing to add here, write "See above."*
Recruitment	*Describe how participants were recruited. Outline any potential self-selection bias or other biases that may be present and how these are likely to impact results.*
Ethics oversight	*Identify the organization(s) that approved the study protocol.*

Note that full information on the approval of the study protocol must also be provided in the manuscript.

Field-specific reporting

Please select the one below that is the best fit for your research. If you are not sure, read the appropriate sections before making your selection.

☒ Life sciences ☐ Behavioural & social sciences ☐ Ecological, evolutionary & environmental sciences

For a reference copy of the document with all sections, see nature.com/documents/nr-reporting-summary-flat.pdf

Life sciences study design

All studies must disclose on these points even when the disclosure is negative.

Sample size	No sample size calculation was performed. For cell wall biochemistry experiments, each sample of Arabidopsis inflorescence stems comprised at least from 6-9 individuals per genotype. This is determined by availability of space in growth chambers and previous determination of variability in cell wall composition between individuals.
Data exclusions	There were no data exclusions.

Replication	In vitro GalT-assay with pine deacetylated AcGGM and Arabidopsis seed mucilage beta-GGM were performed at least twice with the different tobacco membrane preparation. The assay of CtMAGT on ivory nut homomannan was performed twice independently. For the assay on pine AcGGM ANTS-labelled mannohexaose, a second fully independent replicate was not attempted.<br>Three biological replicates from plants grown at different times were used for all cell wall biochemistry experiments.<br>For the cellulose adsorption assay, cell wall fractions from three biological replicates were used with three technical replicates. All attempts at replication were successful.
Randomization	Plants were grouped by genotype, and control wild types were grown at the same time in adjacent trays in the growth rooms.
Blinding	Microscopic observation for Arabidopsis seed mucilage was also conducted by a blinded investigator. None of the other experiments included a subjective element; therefore, blinding was irrelevant.

Reporting for specific materials, systems and methods

We require information from authors about some types of materials, experimental systems and methods used in many studies. Here, indicate whether each material, system or method listed is relevant to your study. If you are not sure if a list item applies to your research, read the appropriate section before selecting a response.

Materials & experimental systems

n/a	Involved in the study	
☐	☒	Antibodies
☒	☐	Eukaryotic cell lines
☒	☐	Palaeontology and archaeology
☒	☐	Animals and other organisms
☒	☐	Clinical data
☒	☐	Dual use research of concern
☐	☒	Plants

Methods

n/a	Involved in the study	
☒	☐	ChIP-seq
☒	☐	Flow cytometry
☒	☐	MRI-based neuroimaging

Antibodies

Antibodies used	Rabbit anti-Myc antibody (1:2000, lot# 1007474-2, cat# ab9106, Abcam) and goat anti-rabbit IgG hourseradish peroxidase-conjugate (1:10,000, Batch# 64371828, cat #1706515, Bio-Rad) were used for immuno blotting assay to detect Myc-tagged MAGT proteins expressed in the membrane fractions extracted from tobacco leaves.
Validation	Rabbit anti-Myc antibody was validated for immuno blotting by the manufacturer (https://www.abcam.com/en-gb/products/primary-antibodies/myc-tag-antibody-ab9106)

Plants

Seed stocks	T-DNA insertion line (SALK_061576) of magt1-1 Arabidopsis mutant was obtained from NASC (https://arabidopsis.info/).
Novel plant genotypes	Transgenic Arabidopsis plants (pIRX3::AtMAGT1-Myc, pIRX3::AtMAGT2-Myc, pIRX3::PtMAGT-Myc, pIRX3::CtMAGT-Myc under wild-type background, and pAtMAGT1::AtMAGT1-Myc, pAtMAGT1::AtMAGT2-Myc, pAtMAGT1::PtMAGT-Myc, pAtMAGT1::CtMAGT-Myc under magt1-1 background) were generated by agrobacterium-mediated floral dipping. At least five independent lines for each genotype were tested for cell wall analysis and three independent lines were selected. T3 generation of homozygous lines was used for all analyses.
Authentication	Three independent transgenic plants exhibited no phenotypical difference and showed the same biochemical effects.
